# Chrysin Inhibits NF-κB-Dependent *CCL5* Transcription by Targeting IκB Kinase in the Atopic Dermatitis-Like Inflammatory Microenvironment

**DOI:** 10.3390/ijms21197348

**Published:** 2020-10-05

**Authors:** Hyunjin Yeo, Young Han Lee, Dongsoo Koh, Yoongho Lim, Soon Young Shin

**Affiliations:** 1 Department of Biological Sciences, Sanghuh College of Lifesciences, Konkuk University, Seoul 05029, Korea; jini1606@konkuk.ac.kr (H.Y.); yhlee58@konkuk.ac.kr (Y.H.L.); 2 Cancer and Metabolism Institute, Konkuk University, Seoul 05029, Korea; 3 Department of Applied Chemistry, Dongduk Women’s University, Seoul 02748, Korea; dskoh@dongduk.ac.kr; 4 Division of Bioscience and Biotechnology, BMIC, Konkuk University, Seoul 05029, Korea; yoongho@konkuk.ac.kr

**Keywords:** atopic dermatitis, CCL5, chemokine, chrysin, mast cell

## Abstract

Chrysin (5,7-dihydroxyflavone) is a natural polyphenolic compound that induces an anti-inflammatory response. In this study, we investigated the molecular mechanism underlying the chrysin-induced suppression of C-C motif chemokine ligand 5 (*CCL5*) gene expression in atopic dermatitis (AD)-like inflammatory microenvironment. We showed that chrysin inhibited *CCL5* expression at the transcriptional level through the suppression of nuclear factor kappa B (NF-κB) in the inflammatory environment. Chrysin could bind to the ATP-binding pocket of the inhibitor of κB (IκB) kinase (IKK) and, subsequently, prevent IκB degradation and NF-κB activation. The clinical efficacy of chrysin in targeting IKK was evaluated in 2,4-dinitrochlorobenzene-induced skin lesions in BALB/c mice. Our results suggested that chrysin prevented *CCL5* expression by targeting IKK to reduce the infiltration of mast cells to the inflammatory sites and at least partially attenuate the inflammatory responses. These findings suggested that chrysin might be useful as a platform for the design and synthesis of small-molecule IKK-targeting drugs for the treatment of chronic inflammatory diseases, such as AD.

## 1. Introduction

Atopic dermatitis (AD) is a chronic inflammatory skin disease that commonly occurs in children. The cause of AD is yet to be fully elucidated; however, the onset of AD is associated with genetic and environmental factors, skin architectural defects, and cell-mediated immune dysfunction [1]. T-helper cell (Th2)-predominant inflammatory responses are believed to promote AD pathogenesis and immunoglobulin E (IgE)-mediated hypersensitivity [2,3], and these are often associated with intractable chronic itchiness [4]. The chronicity of itching and scratching is a common symptom of AD, which reduces the quality of life for patients.

Mast cells are multifunctional immune cells that link innate and adaptive immunity and play a major role in immunoglobulin E (IgE)-mediated hypersensitivity in AD [5]. Activated mast cells produce various inflammatory mediators, including histamine; lipid mediators, such as prostaglandins; growth factors; cytokines; and chemokines, such as tumor necrosis factor alpha (TNFα), interleukin (IL)-1β, IL-4, and IL-6, which are associated with the pathogenesis of AD [5]. The number of mast cells increases in most patients with AD and skin lesions in mouse AD models, which implies that mast cells are involved in the incidence and severity of AD [6].

The inflammatory chemokine C-C motif chemokine ligand 5 (CCL5), also known as regulated on activation, normal T cell expressed and secreted (RANTES), belongs to the C-C chemokine family and plays an active role in directing mast cells to inflammatory sites [7]. CCL5 is overexpressed in the skin of patients with AD [8], and CCL5 antagonism has shown therapeutic efficacy in models of contact skin inflammation [9]. CCL5 expression is regulated in a cell-type- and stimulus-dependent manner by several transcription factors, including nuclear factor kappa B (NF-κB), Activator protein 1 (AP1), Nuclear factor for IL6 expression (NF-IL6), Specificity protein 1 (SP1), and Krüppel-like factor 13 [10,11].

**Figure 1:**
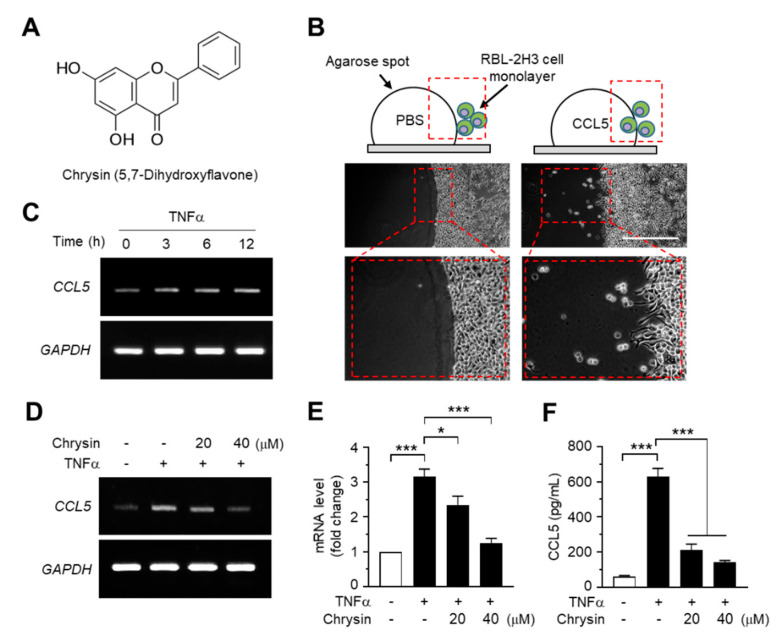
Effects of chrysin on the inhibition of C-C motif chemokine ligand 5 (CCL5) expression. (**A**) Chemical structure of chrysin (5,7-dihydroxyflavone). (**B**) Agarose spot migration assay. The RBL2H3 cell suspension was plated on an agarose spot containing phosphate-buffered saline (PBS) or 25-ng/mL CCL5. At 6 h after the addition of PBS or CCL5, the chemotactic cells were imaged. The areas in the dashed boxes are enlarged in the bottom panels. (**C**) HaCaT cells were treated with 10-ng/mL tumor necrosis factor alpha (TNFα) for 0, 3, 6, and 12 h. Total RNA was isolated, and the levels of *CCL5* mRNA were measured using reverse transcription (RT)-PCR. Glyceraldehyde 3-phosphate dehydrogenase (*GAPDH*) mRNA levels were measured as an internal control. (**D**) HaCaT cells were pretreated with 20 and 40-μM chrysin for 30 min before stimulation with 10-ng/mL TNFα. After 12 h, total RNA was isolated, and the levels of *CCL5* mRNA were measured using RT-PCR. *GAPDH* mRNA levels were measured as an internal control. (**E**) HaCaT cells were treated as in (**D**), and total RNA was isolated. The expression level of *CCL5* mRNA was quantified using quantitative (Q)-PCR with SYBR^TM^ Green-based fluorescent probes. The relative expression fold was normalized to the levels of *GAPDH*. Data are expressed as the mean ± SD (*n* = 3). * *p* = 0.0111 and *** *p* < 0.001 by Dunnett’s multiple comparisons test. (**F**) HaCaT cells were treated with chrysin and TNFα, as in (**E**). After 24 h, the culture medium was collected, and CCL5 protein levels were measured using the enzyme-linked immunosorbent assay (ELISA). Data are expressed as the mean ± SD (*n* = 3). *** *p* < 0.001 by Dunnett’s multiple comparisons test.

Chrysin (5,7-dihydroxyflavone, Figure 1A) is a polyphenolic flavonoid compound that is abundant in honey, propolis, and carrots. It exhibits multiple biological properties, including anti-inflammatory and anticancer properties [12]. Chrysin has been shown to inhibit allergen-induced [13] and TNFα-induced NF-κB activity [14,15] and alleviate AD-like skin lesions in a mouse model [15]. However, the molecular mechanisms underlying the suppression of CCL5 expression and inhibition of NF-κB activity by chrysin remain unknown.

In this study, we found that chrysin is able to bind to the ATP-binding pocket of the inhibitor of κB (IκB) kinase (IKK), consequently inhibiting the IKK kinase activity and downregulating the NF-κB signaling pathway, thereby inhibiting the transcription of CCL5 at the gene promoter level.

## 2. Results and Discussion

### 2.1. Effect of Chrysin on the Suppression of TNFα-Induced CCL5 Expression in HaCaT Cells

As CCL5 was observed to enhance the motility of eosinophilic RBL2H3 leukemia cells in the agarose spot migration assay (Figure 1B), we reasoned that chrysin exhibits anti-inflammatory activity through the modulation of CCL5, at least partially. To test this theory, we evaluated the effects of chrysin treatment on *CCL5* mRNA expression in HaCaT keratinocytes, a human skin equivalent cell model. TNFα is a proinflammatory cytokine that plays a major role in the pathogenesis of AD [16]. Reverse transcription (RT)-PCR analyses showed that TNFα elevated *CCL5* mRNA expression in a time-dependent manner (Figure 1C), whereas the pretreatment with chrysin reduced TNFα-induced *CCL5* mRNA expression in HaCaT cells (Figure 1D). The change in mRNA levels was quantified by quantitative PCR (Q-PCR) with SYBR^TM^ Green-based fluorescent probes specific for *CCL5*. TNFα increased *CCL5* mRNA levels by 3.16 ± 0.212-fold; however, in the presence of 20 and 40-μM chrysin, the *CCL5* mRNA levels, which increased under TNFα induction, decreased by 2.35 ± 0.250- and 1.24 ± 0.144-fold, respectively, compared to those in the control (Figure 1E). Consistently, findings from the enzyme-linked immunosorbent assay (ELISA) of the HaCaT-conditioned medium showed that chrysin significantly (*p* < 0.001, *n* = 3) reduced the TNFα-induced accumulation of CCL5 (Figure 1F).

### 2.2. Effect of Chrysin on the Inhibition of TNFα-Induced CCL5 Promoter Activity

**Figure 2:**
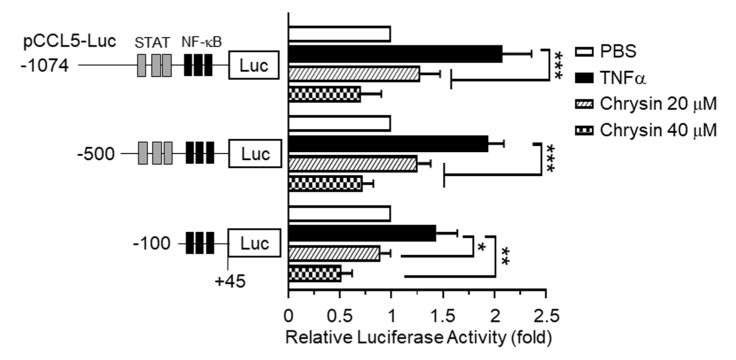
Effect of chrysin on the inhibition of TNFα-induced CCL5 promoter activity. HaCaT cells were transfected with 0.2 µg of a set of 5′-deletion constructs of CCL5 promoter-reporter plasmids. At 48 h post-transfection, the cells were treated with 10-ng/mL TNFα in the absence or presence of chrysin (20 or 40 μM). After 12 h, the cells were harvested, and luciferase reporter activities were measured. The schematic diagram shows a set of deletion constructs of the CCL5 promoter-reporter plasmid. Putative Signal Transducer and Transcription (STAT) and nuclear factor kappa B (NF-κB) binding sites are indicated by grey and black boxes, respectively. Data are expressed as the mean ± SD (*n* = 3). * *p* = 0.0134, ** *p* = 0.0016, and *** *p* < 0.0001 by Sidak’s multiple comparisons test.

The 5’-regulatory region of human *CCL5* contains multiple *cis*-acting regulatory elements, including the NF-κB and Signal Transducer and Transcription (STAT) binding sites, and is regulated in a cell-type- and stimulus-specific manner [10]. To determine whether chrysin inhibits *CCL5* expression at the transcriptional level, we isolated the 5′-regulatory region of *CCL5* spanning nucleotides –1074 to +45 and generated a set of deletion constructs, –1074/+45, –500/+45, and –100/+45, containing a luciferase reporter enzyme. Each of these promoter-reporter constructs was transiently transfected into HaCaT cells, and the levels of luciferase activity were evaluated. We observed that, after TNFα stimulation, the *CCL5* promoter-reporter activity persisted in even the shortest reporter (–93 to +65) containing a cluster of NF-κB-binding elements, which was subsequently inhibited by chrysin (Figure 2). These results suggest that chrysin inhibits *CCL5* expression at the gene promoter level and that NF-κB participates in the chrysin-induced suppression of *CCL5* transcription.

### 2.3. Binding of Chrysin to the ATP-Binding Pocket of IKK

**Figure 3:**
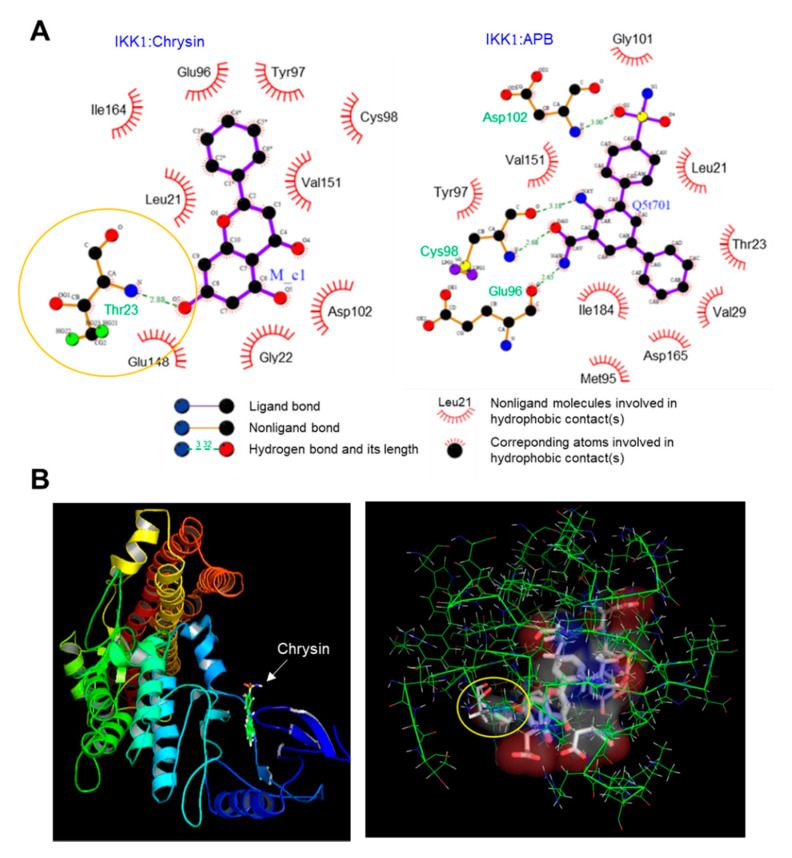
Molecular docking simulation between the inhibitor of κB kinase 1 (IKK1) and chrysin. (**A**) The interactions between the IKK1 protein and chrysin (**left**) and the reference ligand, 2-azanyl-5-phenyl-3-(4-sulfamoylphenyl)benzamide (APB) (**right**), were analyzed using LigPlot. (**B**) The 3D images of the IKK1:chrysin complex (**left**) and the binding pocket of the chrysin:IKK1 complex (**right**) generated using PyMOL. The distance between the 7-hydroxyl group of chrysin and nitrogen of Thr23 that forms an H-bond is 2.88 Å (indicated by the yellow circle).

Chrysin is known to inhibit NF-κB [13,14]; however, the mechanism underlying the inhibition of NF-κB activity by chrysin has not been reported to date. As the phosphorylation of IκB by IKK is the key step in the activation of NF-κB, we hypothesized that chrysin might modulate the IKK activity. To evaluate this, we performed an in silico molecular docking experiment to predict the possible binding mode of chrysin to IKK. We used the LigPlot program to analyze the binding sites. Thirty protein-ligand complexes were generated. The binding energy ranged from −19.3 to −13.0 kcal/mol. The complex with the lowest binding energy was selected and analyzed using LigPlot. Nine residues, including Leu21, Gly22, Glu96, Tyr97, Cys98, Asp102, Glu148, Val151, and Ile164, participated in the hydrophobic interaction with chrysin, and a residue, Thr23, formed a hydrogen bond (H-bond) (Figure 3A, left). The distance between the 7-hydroxyl group of chrysin and nitrogen of Thr23 that formed an H-bond was 2.88 Å (indicated by the yellow circle). Notably, chrysin was docked in the same ATP-binding pocket of the reference ligand, 2-azanyl-5-phenyl-3-(4-sulfamoylphenyl)benzamide (APB) and IKK1 (Figure 3A, right). The 3D images for the chrysin-IKK1 complex were generated using PyMOL (Figure 3B). This observation suggests that chrysin can bind to the ATP-binding pocket of IKK, which leads to the inhibition of the NF-κB signaling pathway.

### 2.4. Effect of Chrysin on the Inhibition of the IKK Downstream Pathway

**Figure 4:**
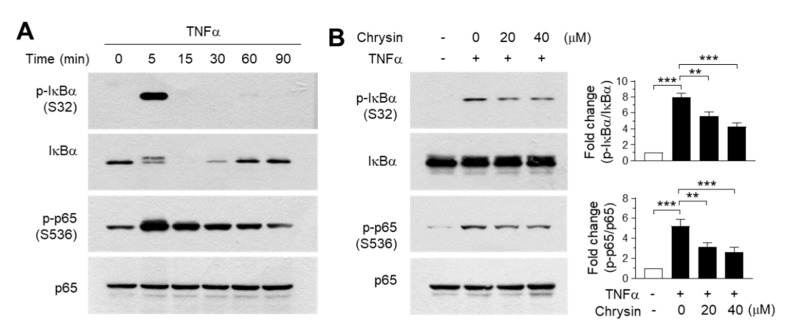
Effect of chrysin on the inhibition of the downstream signaling pathway of the inhibitor of κB (IκB) kinase (IKK). (**A**) HaCaT cells were treated with 10-ng/mL TNFα for different periods (0–90 min), and the phosphorylation of IκB at Ser32 and p65/RelA NF-κB at Ser536 were observed in immunoblotting experiments. Total IκB and p65 proteins were used as the internal controls. (**B**) HaCaT cells were pretreated with chrysin (20 and 40 μM) for 30 min, then stimulated with 10-ng/mL TNFα for 5 min. The phosphorylation of IκB and p65/RelA NF-κB were measured by immunoblot analysis. The band intensity corresponding to each phosphorylated protein was normalized to the total protein levels for each protein using ImageJ software. The bars represent mean ± SD (*n* = 3). ** *p* = 0.0005 and *** *p* < 0.0001 by Dunnett’s multiple comparisons test.

To validate the findings of the molecular docking experiments, we examined the inhibitory effect of chrysin on the IKK downstream signaling pathway. TNFα was observed to induce the phosphorylation of IκB at Ser32 and p65/RelA at Ser536 within 5 min of treatment (Figure 4A). Notably, IκB was almost completely degraded to undetectable levels within 15 min—after which, the levels were gradually restored. When the cells were pretreated with 20 and 40-μM chrysin for 30 min before stimulation with 10-ng/mL TNFα for 5 min, TNFα-induced IκB and p65/RelA NF-κB phosphorylation reduced significantly (all *p* < 0.01, *n* = 3) in a dose-dependent manner (Figure 4B). Therefore, chrysin suppresses TNFα-induced NF-κB activation in HaCaT keratinocytes. Although we did not evaluate the effect of chrysin on the inhibtion of IKK downstream pathways in primary keratinocytes, it has been reported that chrysin inhibits TNFα plus the interferon gamma (IFNγ)-induced phosphorylation of NF-κB in primary keratinocytes [15], suggesting that chrysin attenuates IKK downstream signaling pathways by targeting IKK in skin keratinocytes.

### 2.5. Effect of Chrysin on the Inhibition of NF-κB in 2,4-Dinitrochlorobenzene (DNCB)-Induced Skin Lesions

**Figure 5:**
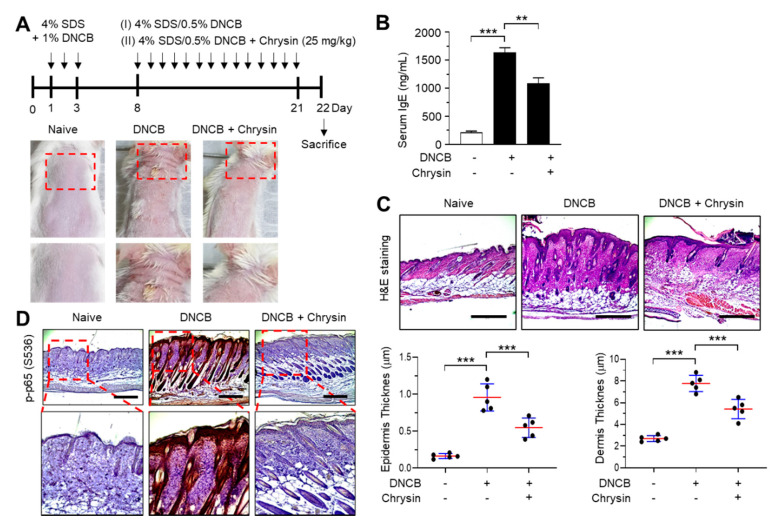
Effect of chrysin on the inhibition of NF-κB in 2,4-dinitrochlorobenzene (DNCB)-induced skin lesions. (**A**) Illustration of the experimental schedule for the induction of atopic dermatitis (AD)-like skin lesions and treatment with chrysin (top). Representative images depicting the phenotype of naïve, DNCB-induced skin lesions, and DNCB + chrysin (25 mg/kg)-treated skin in BALB/c mice (bottom). The areas in the dashed boxes are enlarged in the bottom panels. (**B**) Blood samples were collected immediately before sacrifice on day 22, and the total serum immunoglobulin E (IgE) levels were measured by the enzyme-linked immunosorbent assay. Data are expressed as the mean ± SD (*n* = 3). ** *p* = 0.0002 and *** *p* < 0.0001 by Dunnett’s multiple comparisons test. (**C**) Histological analysis. Paraffin-embedded skin sections were stained with hematoxylin and eosin (H&E). Scale bar, 400 μm. The thicknesses of the epidermis and dermis were measured using ImageJ. Data are expressed as the mean ± SD (*n* = 3). *** *p* < 0.001 by Dunnett’s multiple comparisons test. (**D**) Immunohistochemical staining of phosphorylated p65 (p-p65) in Ser536 in skin tissues treated with DNCB and DNCB + 0.2% chrysin. The sections were counterstained with H&E. Scale bar, 400 μm. The areas in the dashed boxes are enlarged in the bottom panels.

The efficacy of chrysin in IKK inhibition in vivo was evaluated in BALB/c mice repeatedly challenged with 2,4-dinitrochlorobenzene (DNCB) on the dorsal skin. Mice that were challenged with DNCB for 21 days developed AD-like skin lesions, and the topical application of chrysin (25 mg/kg) improved this clinical symptom (Figure 5A). Increased serum IgE levels are an important diagnostic indicator of DNCB-induced AD-like skin lesions [17]. We measured the serum immunoglobulin E (IgE) levels in blood samples collected from mice before sacrifice. The topical application of chrysin significantly (*p* < 0.001, *n* = 5) decreased the serum IgE levels compared to those in DNCB-treated mice (Figure 5B). A histopathological analysis, which involved hematoxylin and eosin (H&E) staining, and a morphometric analysis showed that the DNCB-induced increase in epidermal and dermal thickenings reduced significantly (*p* < 0.001, *n* = 5) upon the topical application of chrysin (Figure 5C). Previous studies have reported that DNCB increases the phosphorylation of p65 NF-κB in BALB/c mice models [18,19]. We also observed that the epidermal levels of phosphorylated p65 NF-κB were high in mice challenged with DNCB; however, the levels decreased substantially upon the topical application of chrysin (Figure 5D). These results suggest that IKK targeting by chrysin could lead to the inhibition of NF-κB in vivo.

### 2.6. Effect of Chrysin on the Suppression of CCL5 Expression and Infiltration of Mast Cells in DNCB-Induced Skin Lesions

**Figure 6:**
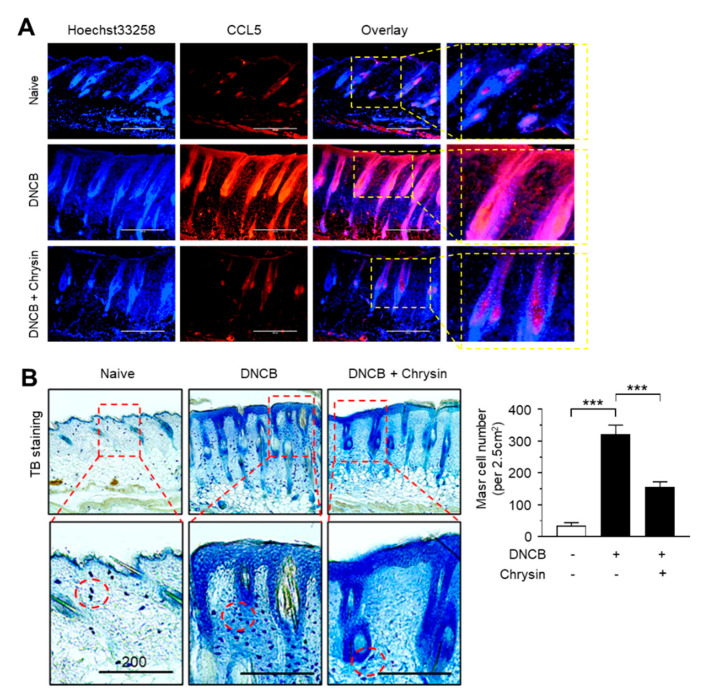
Effect of chrysin on the suppression of CCL5 expression and the infiltration of mast cells in DNCB-induced skin lesions. (**A**) Immunofluorescence staining of CCL5 using rhodamine red-X-conjugated secondary antibody (red) in skin tissues treated with DNCB and DNCB + 0.2% chrysin. Nuclei were counterstained using Hoechst 33258 (blue). Scale bar, 400 μm. Magnified view of the areas in the dashed boxes are provided in the right panels. (**B**) Mast cells were stained with 0.1% toluidine blue in skin tissues treated with DNCB and DNCB + chrysin (25 mg/kg). The areas in the dashed boxes are enlarged in the bottom panels. The dotted circles indicate the infiltrating mast cells (blue spots). Scale bar, 200 μm. The number of mast cells per 2.5 cm^2^ of area was counted. Graph data are expressed as the mean ± SD (*n* = 5). *** *p* < 0.0001 by Dunnett’s multiple comparisons test.

Next, we visualized the expression pattern of CCL5 in mouse skin tissues by immunofluorescence staining. Notably, CCL5-positive staining increased considerably in the epidermis of mice challenged with DNCB, which reduced substantially upon the topical application of chrysin (Figure 6A). It has been reported that chrysin inhibits mast cell-derived allergic inflammation [13]. To determine whether the chrysin-induced suppression of *CCL5* expression affects the infiltration of inflammatory cells, we performed mast cell staining in DNCB-challenged skin tissue sections using toluidine blue (TB). In accordance with a previous study [19], DNCB increased the infiltration of TB-positive mast cells; however, this recruitment of TB-positive cells by DNCB was reduced significantly (*p* < 0.001, *n* = 5) upon the repeated application of chrysin (Figure 6B). These results suggest that IKK targeting by chrysin led to the suppression of NF-κB activity and reduced NF-κB-regulated CCL5 expression, subsequently suppressing the inflammatory responses and infiltration of inflammatory cells, such as mast cells, in DNCB-induced skin lesions in BALB/c mice.

## 3. Materials and Methods

### 3.1. Materials

Chrysin, 2,4-dinitrochlorobenzene (DNCB), toluidine blue (TB), and hematoxylin and eosin (H&E) staining kits were obtained from Sigma-Aldrich (St. Louis, MO, USA). Low-melting agarose was purchased from Lonza (Rockland, ME, USA). CCL5 was obtained from PeproTech (London, UK). TNFα was obtained from ProSpec-Tany TechnoGene, Ltd. (Ness-Ziona, Israel). A Firefly Luciferase Assay System was obtained from Promega (Madison, WI, USA). Anti-CCL5 antibody was obtained from Invitrogen (Thermo Fisher Scientific, Waltham, MA, USA), and anti-IKKα/β, -IκB, -phospho-IκB (Ser32), -p65/RelA, and -phospho-p65/RelA (Ser536) antibodies were obtained from Cell Signaling Technology (Danvers, MA, USA). Secondary antibody conjugated to rhodamine red-X was obtained from Jackson ImmunoResearch Laboratories (West Grove, PA, USA).

### 3.2. Cells and Cell Culture

Human keratinocyte HaCaT cells were obtained from Cell Lines Service (Eppelheim, Germany) and maintained in Dulbecco’s modified Eagle’s medium supplemented with 10% fetal bovine serum (HyClone, Logan, UT, USA) and penicillin-streptomycin (Sigma-Aldrich, St. Louis, MO, USA).

### 3.3. Reverse Transcription-PCR (RT-PCR)

Total RNA was isolated using a TRIzol RNA extraction kit (Invitrogen, Carlsbad, CA, USA). cDNA was synthesized from 1 μg of total RNA using an iScript cDNA synthesis kit (Bio-Rad, Hercules, CA, USA). RT-PCR was performed using a reverse transcriptase enzyme (Promega) and gene-specific PCR primers. The PCR primers and the thermal cycling conditions used in this study were as follows:

- *CCL5* forward, 5′-ACA GGT ACC ATG AAG GTC TC–3′,
- *CCL5* reverse, 5′–GCA AAT TTG TGT AAG TTC AGG–3,
- Glyceraldehyde 3-phosphate dehydrogenase (*GAPDH*) forward, 5′–CCA AGG AGT AAG AAA CCC TGG AC–3′, and
- *GAPDH* reverse, 5′–GGG CCG AGT TGG GAT AGG G–3′.

The PCR conditions were as follows: denaturation at 94 °C (5 min), followed by 30 cycles comprising denaturation at 94 °C (30 s), annealing at 58 °C (30 s), and elongation at 72 °C (1 min). The amplified products were electrophoresed in a 2% agarose gel containing ethidium bromide and observed under UV light.

### 3.4. Quantitative Real-Time PCR (Q-PCR)

The mRNA levels were quantified using an iCycler iQ system with an iQ SYBR Green Supermix kit (Bio-Rad, Hercules, CA, USA). Validated commercial Q-PCR primers and SYBR Green-based fluorescent probes specific for *CCL5* (id: qHsaCIP0028116) and *GAPDH* mRNA (id: qHsaCEP0041396) were obtained from Bio-Rad (Hercules, CA, USA). The thermal cycling conditions used for PCR were as follows: denaturation at 95 °C for 2 min, followed by 40 cycles performed using a step program (95 °C for 10 s and 60 °C for 45 s). The relative expression levels of *CCL5* mRNA were normalized to those of *GAPDH* using the software provided by the manufacturer.

### 3.5. Agarose Spot Migration Assay

Chemotactic migration was analyzed in the agarose spot migration assay according to a process described previously [20], with minor modifications introduced. Low-melting agarose (0.5%) in PBS was heated in a microwave and stirred for complete dissolution. When the agarose particles completely dissolved, the liquid agarose solution was cooled to 40 °C, followed by mixing with only PBS or PBS supplemented with CCL5 (final: 25 ng/mL) in a 1.5-mL Eppendorf tube. Next, 10-μL drops of agarose solution containing PBS or CCL5 were placed on each sterile glass coverslip in a 6-well plate using a cut pipette tip and were allowed to cool for 10 min at 4 °C to solidify the agar spots. A suspension of RBL2H3 cells in PBS was plated on the spot-containing coverslips and allowed to adhere for 4 h in an incubator at 37 °C. The cells were incubated overnight at 37 °C with a culture medium containing 0.1% fetal bovine serum. After 6 h, the motile cells that penetrated the agarose spot were analyzed under a microscope (EVOS FL Auto, Bothell, WA, USA).

### 3.6. Enzyme-Linked Immunosorbent Assay (ELISA)

CCL5 secreted in the culture medium was quantified using an ELISA kit (Biolegend, London, UK) according to the manufacturer’s instructions. In brief, the captured antibodies were coated on a Nunc C bottom immunoplate. The wells were washed three times with 50-mM Tris buffer (pH 8.0) containing 0.14-M NaCl and 0.05% Tween 20 (TBST). The collected culture supernatants and standard solutions were added to the wells, and the plates were incubated at 37 °C for 2 h. The wells were washed three times with TBST solution, 50 μL of biotin-conjugated anti-CCL5 antibody (1:200) was added, and the cultures were incubated at 37 °C for 1 h. After washing five times with TBST solution, horseradish peroxidase-conjugated tracer antibody was added to each well, and the cultures were incubated for an additional 1 h. An enzymatic reaction was initiated by adding tetramethylbenzidine substrate solution (containing 100-mM sodium acetate buffer (pH 6.0) and 0.006% H_2_O_2_), followed by incubation at 37 °C for 20 min. The reaction was terminated by adding an acidic solution (reaction stopper, 1-M H_2_SO_4_), and the absorbance was measured at 450 nm using an ELISA plate reader (SoftMax Pro; Molecular Devices, Sunnyvale, CA, USA). The final concentration of CCL5 was calculated using the standard curve.

### 3.7. Construction of Human CCL5 Promoter-Reporter Constructs

A *CCL5* promoter fragment spanning nucleotides –1074 to +45 upstream of the transcription start site was synthesized from human genomic DNA (Promega, Madison, WI, USA) by PCR using the primers 5′-GAG GGC AAC TGG GTT CTG AT-3′ (forward –1074F) and 5′-GAG GTC CAC GTG CTG TCT TG-3′ (reverse, +45R). The amplified PCR products were ligated to a T&A vector (RBC Bioscience, Taipei County, Taiwan) and digested with *Kpn*I and *Bgl*II. The products were ligated at the *Kpn*I and *Bgl*II sites of the pGL4 basic vector (Promega), yielding pCCL5-Luc(–1074/+45). Several deletion constructs of the human *CCL5* promoter fragments were synthesized by PCR using the pCCL5-Luc(–1074/+45) construct as a template plasmid. The forward primer sequences were 5′-TGA GTG TGC TCA CCT CCT TT-3′ (−500/+45) and 5′-TGT GCA ATT TCA CTT ATG AT-3′ (–100/+45). One reverse primer, +45R, was used to generate all the deletion constructs. The amplified PCR products were ligated into the T&A vector and then digested using *Kpn*I and *Bgl*II. The products were ligated into the *Kpn*I and *Bgl*II sites of the pGL4 basic vector. The insert sequence of each construct was verified by DNA sequencing (Macrogen, Seoul, Republic of Korea).

### 3.8. Luciferase Promoter-Reporter Assay

HaCaT cells were seeded onto 12-well plates and transfected with 0.1 µg of each *CCL5* promoter-reporter construct using Lipofectamine^™^ 2000 (Invitrogen) according to the manufacturer’s instructions. At 48 h post-transfection, the cells were treated with TNFα in the presence or absence of chrysin (20 and 40 μM). After 8–12 h, the cells were harvested, and the levels of firefly luciferase activity were measured using the Dual-Glo^TM^ Luciferase assay system (Promega). The relative level of luciferase activity in the untreated cells was designated 1. Luminescence was measured using a dual luminometer (Centro LB960; Berthold Tech, Bad Wildbad, Germany).

### 3.9. Immunoblot Analysis

HaCaT cells were lysed in ice-cold buffer containing 50-mM Tris-HCl (pH 7.4), 1% NP-40, 0.25% Na-deoxycholate, 500-mM NaCl, 1-mM ethylenediaminetetraacetic acid, 1 mM-Na_3_VO_4_, 1-mM NaF, 10-μg/mL leupeptin, and 1-mM phenylmethylsulfonyl fluoride. Proteins were separated by electrophoresis in a 10% SDS-polyacrylamide gel and transferred to nitrocellulose membranes. After incubation with the appropriate primary and secondary antibodies, the blots were developed using an enhanced chemiluminescence detection system (GE Healthcare, Piscataway, NJ, USA).

### 3.10. Molecular Docking

To elucidate the binding modes between chrysin and IKK1, in silico docking experiments were performed using the Sybyl 7.3 software (Tripos, St. Louis, MO, USA) built on an Intel Core 2 Quad Q6600 (2.4 GHz) Linux PC. There are five three-dimensional (3D) structures of IKK1 deposited in the Protein Data Bank (PDB): 3brt, 5ebz, 5tqw, 5tqx, and 5tqy. One of them, 3brt.pdb, only includes residues between 732 and 745, owing to which, it could not be considered for constructing the 3D structure for use in the current study. The others include residues from 10–660. While the structures 5ebz, 5tqw, and 5tqx were determined by cryoEM, and their resolution was 5.60 Å, 5ebz.pdb was determined by X-ray crystallography, and its resolution was 4.50 Å. Therefore, 5ebz.pdb was selected for constructing the 3D structure in this study [21]. Its gene originated in *Homo sapiens*, and *Spodoptera frugiperda* was used as the expression system. It consists of a trimer of IKK1 dimers; chain A (Gly10–Glu660) was selected for the docking experiment, and 2-azanyl-5-phenyl-3-(4-sulfamoylphenyl)benzamide was used as a reference ligand. The binding site was analyzed using the LigPlot program provided by the European Bioinformatics Institute (Cambridgeshire, UK) [22]. The apo-protein without the ligand was prepared using Sybyl 7.3. The chrysin 3D structure deposited in PubChem (CID code 5281607, National Center for Biotechnology Information, Bethesda, MD, USA) was used after energy minimization using the molecular mechanics algorithms provided in Sybyl 7.3. All 3D images were constructed using PyMOL (The PyMOL Molecular Graphics System, Version 1.0r1, Schrödinger, LLC, New York, NY, USA).

### 3.11. Mice

BALB/c mice (7-week-old, male) were obtained from Orient Bio, Inc. (Seongnam, Korea). The mice were housed in a specific pathogen-free environment at a temperature of 20 ± 2 °C and relative humidity of 50% ± 10% and were maintained under a 12-h light-12-h dark cycle.

### 3.12. Induction of Atopic Dermatitis-like Skin Lesions in Mice

DNCB was dissolved in a 1:3 (v/v) mixture of acetone:olive oil. After the dorsal skin was shaved, BALB/c mice were randomly divided into three groups: group I, naïve, group II, DNCB + vehicle, and group III, DNCB + 8% chrysin (*n* = 5 each). All mice, except those in the naïve group, were treated with 4% SDS on the dorsal skin to disrupt the skin barrier. After 4 h, 100 μL of 1% DNCB was challenged once daily, and this was repeated for 3 days. After a 4-day rest period, a treatment with 100 μL of 4% SDS and 0.5% DNCB was repeated once daily, five times weekly, for 2 weeks (from days 8–21). Chrysin powder (250 mg) was dissolved in 1 mL of dimethyl sulfoxide to prepare a stock solution. Group III mice were applied with chrysin (25 mg/kg) from day 7 (once daily, five times weekly for 2 weeks). The animal experiments were conducted according to the guidelines for animal experiments and procedures approved by the Konkuk University Institutional Animal Care and Use Committee (IACUC, Seoul, Republic of Korea), and all experimental methods were confirmed to be in accordance with the relevant guidelines and regulations (approval number KU19129).

### 3.13. Tissue Preparation And Histopathological Analysis

Dorsal skin tissues of mice were removed, fixed with 100% acetone solution, and embedded in paraffin. Skin sections 5 μm in thickness were cut using a microtome (Leica Microsystems, Wetzlar, Germany). After dewaxing, the sections were stained with H&E. Images of each section were acquired using a light microscope (EVOS FL Auto, Bothell, WA, USA), and the epidermal and dermal thicknesses were measured from the digital images using ImageJ 1.52a (National Institutes of Health, Bethesda, MD, USA). The infiltrating mast cells were stained with 0.1% TB. The number of TB-positive cells in 2.5 cm^2^ was counted.

### 3.14. Immunohistochemical and Immunofluorescence Stainings

Paraffin-embedded dorsal skin sections were deparaffinized using xylene and rehydrated by treating with a graded ethanol series. Skin specimens were treated with hydrogen peroxide for 15 min to block endogenous peroxidase activity, followed by immersion in 1-mM EDTA (pH 8.0) at 70 °C for 20 min. After rinsing with PBS, the sections were placed in a blocking buffer containing 7% goat serum for 1 h and incubated with primary anti-p65/RelA antibody overnight at 4 °C. After washing with PBS, the sections were incubated with biotinylated anti-goat IgG secondary antibodies for 1 h and then incubated with an avidin/biotin complex for 30 min. Skin sections were stained with 3,3′-diaminobenzidine tetrahydrochloride for 5 min and counterstained with H&E.

For the fluorescent immunohistochemical analysis, each skin section was treated with a CCL5 antibody (1:100 dilution), followed by overnight incubation at 4 °C. After washing with PBS, the sections were incubated with rhodamine red-X-conjugated secondary antibody (1:300 dilution) at room temperature for 1 h. Nuclei were stained with Hoechst 33258 for 10 min. After washing with PBS, the sections were mounted on slides using a fluorescence mounting medium (ProLong Gold Antifade Reagent; Invitrogen). The fluorescence images were evaluated using an EVOS FL fluorescence microscope (Advanced Microscopy Group, Bothell, WA, USA).

### 3.15. Measurement of Serum IgE Levels

Mouse blood samples were collected before sacrifice, and the serum IgE levels were measured using an ELISA MAX™ Standard Set Mouse IgE Kit (BioLegend, San Diego, CA, USA), as described previously [23]. Color development was quantified by measuring absorbance at 450 nm using an ELISA plate reader (SoftMax Pro; Molecular Devices, Sunnyvale, CA, USA).

### 3.16. Statistical Analysis

Data are expressed as the mean ± standard deviation (SD). Statistical analysis was performed using one-way analysis of variance (ANOVA), followed by Dunnett’s or Sidak’s multiple comparisons tests using GraphPad Prism version 8.4.2 (GraphPad Software, Inc., La Jolla, CA, USA). In all analyses, a *p*-value < 0.05 was considered to indicate statistically significant differences.

## 4. Conclusions

The transcription factor NF-κB is a major regulator of inflammation that is responsible for the expression of multiple inflammatory cytokines and chemokines [24]. The NF-κB family consists of five members: c-Rel, p65/RelA, RelB, p50/NF-κB1, and p52/NF-κB2 and mediates the transcription of various target genes as various homo- or heterodimers [25]. Of these, p65/RelA is the most abundant form. In the resting state, NF-κB is localized in the cytoplasm and remains in an inactive form by binding to the inhibitor of κB (IκB) protein. This interaction prevents the translocation of NF-κB to the nucleus. Following cellular activation, IKK is activated, which consequently phosphorylates IκB, leading to the dissociation of IκB from p65 NF-κB and the eventual activation of NF-κB [26,27]. The activated NF-κB complex is then translocated to the nucleus, where it binds to the NF-κB-binding sites in the promoter region of the regulated genes [28]. Therefore, the phosphorylation of IκB by IKK is critical for the initiation of NF-κB activation [29].

In this study, we observed that chrysin suppresses *CCL5* expression at the transcriptional level by suppressing NF-κB in the inflammatory environment. Using an in silico molecular docking experiment, we predicted that chrysin could bind to the ATP-binding pocket of IKK, which prevents IκB degradation and NF-κB activation. The clinical efficacy of chrysin in targeting IKK was evaluated in DNCB-challenged skin lesions in BALB/c mice. It suppresses CCL5 expression, reduces the infiltration of mast cells into the inflammatory sites, and at least partially alleviates the inflammatory response of inflamed skin challenged with DNCB. Based on these findings, we suggest that chrysin might serve as an IKK inhibitor for the treatment of chronic inflammatory diseases, such as AD. To directly prove the binding between chrysin and IKK, more detailed studies, such as precipitation experiments using agarose-coupled chrysin and surface plasmon resonance experiments, are needed.

In conclusion, this study first demonstrated that chrysin inhibits NF-κB-dependent *CCL5* expression by directly targeting IKK in the AD-like skin inflammatory microenvironment. Our findings contribute to a better understanding of the mechanistic insights into the biological effects of chrysin on anti-inflammatory activity.

## Abbreviations

AD	atopic dermatitis
APB	2-azanyl-5-phenyl-3-(4-sulfamoylphenyl)benzamide
CCL5	C-C motif chemokine ligand 5
DNCB	2,4-dinitrochlorobenzene
ELISA	enzyme-linked immunosorbent assay
GAPDH	glyceraldehyde 3-phosphate dehydrogenase
H&E	hematoxylin and eosin
H-bond	hydrogen bond
IgE	immunoglobulin E
IκB	inhibitor of κB
IKK	inhibitor of κB kinase
IL	interleukin
NF-κB	nuclear factor kappa B
Q-PCR	quantitative real-time PCR
RANTES	regulated on activation, normal T cell expressed and secreted
RelA	v-rel Avian reticuloendotheliosis viral oncogene homolog A
RT-PCR	reverse-transcription polymerase chain reaction
TB	toluidine blue
Th2	T-helper cell (Th2)
TNFα	tumor necrosis factor-alpha
